# Inhibition of diacylglycerol lipase β modulates lipid and endocannabinoid levels in the *ex vivo* human placenta

**DOI:** 10.3389/fendo.2023.1092024

**Published:** 2023-02-14

**Authors:** Natascha Berger, Tom van der Wel, Birgit Hirschmugl, Thomas Baernthaler, Juergen Gindlhuber, Nermeen Fawzy, Thomas Eichmann, Ruth Birner-Gruenberger, Robert Zimmermann, Mario van der Stelt, Christian Wadsack

**Affiliations:** ^1^ Department of Obstetrics and Gynecology, Medical University of Graz, Graz, Austria; ^2^ Department of Molecular Physiology, Leiden Institute of Chemistry, Leiden University and Oncode Institute, Leiden, Netherlands; ^3^ BioTechMed-Graz, Graz, Austria; ^4^ Otto Loewi Research Center, Division of Pharmacology, University of Graz, Graz, Austria; ^5^ Ludwig Boltzmann Institute for Lung Vascular Research, Graz, Austria; ^6^ Diagnostic and Research Center of Molecular Medicine, Diagnostic and Research Institute of Pathology, Medical University of Graz, Graz, Austria; ^7^ Institute of Molecular Biosciences, University of Graz, Graz, Austria; ^8^ Core Facility Mass Spectrometry, Center for Medical Research (ZMF), Medical University of Graz, Graz, Austria; ^9^ Institute of Chemical Technologies and Analytics, Vienna University of Technology, Vienna, Austria

**Keywords:** human placenta, lipid metabolism, endocannabinoid system, 2-arachidonoylglycerol, diacylglycerol lipase, activity-based protein profiling, chemical proteomics, *ex vivo* placental perfusion

## Abstract

**Introduction:**

Lipids and fatty acids are key components in metabolic processes of the human placenta, thereby contributing to the development of the fetus. Placental dyslipidemia and aberrant activity of lipases have been linked to diverse pregnancy associated complications, such as preeclampsia and preterm birth. The serine hydrolases, diacylglycerol lipase α and β (DAGLα, DAGLβ) catalyze the degradation of diacylglycerols, leading to the formation of monoacylglycerols (MAG), including one main endocannabinoid 2-arachidonoylglycerol (2-AG). The major role of DAGL in the biosynthesis of 2-AG is evident from various studies in mice but has not been investigated in the human placenta. Here, we report the use of the small molecule inhibitor DH376, in combination with the ex vivo placental perfusion system, activity-based protein profiling (ABPP) and lipidomics, to determine the impact of acute DAGL inhibition on placental lipid networks.

**Methods:**

DAGLα and DAGLβ mRNA expression was detected by RT-qPCR and in situ hybridization in term placentas. Immunohistochemistry staining for CK7, CD163 and VWF was applied to localize DAGLβ transcripts to different cell types of the placenta. DAGLβ activity was determined by in- gel and MS-based activity-based protein profiling (ABPP) and validated by addition of the enzyme inhibitors LEI-105 and DH376. Enzyme kinetics were measured by EnzChek™ lipase substrate assay. *Ex vivo* placental perfusion experiments were performed +/- DH376 [1 µM] and changes in tissue lipid and fatty acid profiles were measured by LC-MS. Additionally, free fatty acid levels of the maternal and fetal circulations were determined.

**Results:**

We demonstrate that mRNA expression of DAGLβ prevails in placental tissue, compared to DAGLα (p ≤ 0.0001) and that DAGLβ is mainly located to CK7 positive trophoblasts (p ≤ 0.0001). Although few DAGLα transcripts were identified, no active enzyme was detected applying in-gel or MS-based ABPP, which underlined that DAGLβ is the principal DAGL in the placenta. DAGLβ dependent substrate hydrolysis in placental membrane lysates was determined by the application of LEI-105 and DH376. *Ex vivo* pharmacological inhibition of DAGLβ by DH376 led to reduced MAG tissue levels (p ≤ 0.01), including 2-AG (p≤0.0001). We further provide an activity landscape of serine hydrolases, showing a broad spectrum of metabolically active enzymes in the human placenta.

**Discussion:**

Our results emphasize the role of DAGLβ activity in the human placenta by determining the biosynthesis of 2-AG. Thus, this study highlights the special importance of intra-cellular lipases in lipid network regulation. Together, the activity of these specific enzymes may contribute to the lipid signaling at the maternal-fetal interface, with implications for function of the placenta in normal and compromised pregnancies.

## 1 Introduction

The two transmembrane enzymes diacylglycerol lipase alpha (DAGLα) and beta (DAGLβ) possess *sn-1* specific hydrolytic activity for diacylglycerols (DAG), preferably hydrolyzing DAG species with mono- or polyunsaturated fatty acids (FA) at *sn-2* position, leading to the formation of monoacylglycerols (MAG) (1). Interestingly, DAGLα/β exhibit a diverse cell-type and tissue-specific abundancy and it has been shown that specific expression patterns and regulatory mechanisms converge into distinct physiological roles of these enzymes. DAGLα is predominately expressed in the central nervous system and mainly confined to neurons (2, 3). In contrast, DAGLβ is mainly expressed in peripheral tissues where its activity is elevated in immune cells including microglia (3), macrophages (4), dendritic cells (5) and, importantly, associated with inflammatory responses. Besides the constitutive role of DAGLα/β in DAG catabolism, DAGLβ has also been proposed as polyunsaturated fatty acid-specific triacylglycerol lipase (6).

Cell membranes of the human placenta exhibit a high abundance of polyunsaturated arachidonic acid (AA) esterified phosphoglycerides (7) and quantitative analysis of placental lipid profiles revealed a high concentration of unsaturated triacylglycerol species (8). In particular, AA is essentially involved in the development of the fetal brain during the course of pregnancy (9, 10). DAGLα/β are renown as key components of the endocannabinoid system (ECS), regulating the biosynthesis of an AA-esterified monoglyceride, namely 2-arachidonoylglycerol (2-AG). 2-AG is one of the main endocannabinoids, acting as chemical messenger and full agonist for cannabinoid receptors 1 and 2. Both, 2-AG and AA serve as substrates for the synthesis of prostanoid-esters and prostanoids, respectively. These metabolites play an important role during parturition as they mediate processes like contractions of the myometrium and they are involved in a variety of pregnancy pathologies (11–13). The importance to tightly regulate bioactive lipid species is emphasized by several studies reporting aberrant lipase action linked to first trimester miscarriage (14, 15), endometrial cancer (16) and pregnancy associated disorders such as preeclampsia (17, 18). Furthermore, emerging evidence demonstrates a strong association between pregnancy disorders and dyslipidemia (19, 20). Thus, examining one of the key enzymes in lipid metabolism, contributes not only to the basic understanding of the ECS in the human placenta, but more importantly elucidates the potential role of DAGL in pregnancy pathologies.

Although many efforts have been made to describe placental hydrolases in the past decades, many functional aspects are still unknown (21–23). Furthermore, the extent to which DAGL activity may affect metabolic cellular pathways by regulating bioactive lipids in this tissue is not yet understood. Notably, animal models have major limitations to answer this question due to differences in physiology and metabolism of the human placenta compared to other species. In human cytotrophoblasts the presence of DAGLα and the main 2-AG degradative enzyme monoacylglycerol lipase (MGL) has previously been reported (24). Epithelial-like trophoblasts build up the outermost layers of the placenta and are in direct contact with the maternal blood. The multinucleated syncytiotrophoblast derives from underlying cytotrophoblasts and facilitates the exchange of nutrients, wastes and gases between the maternal and fetal circulations. In addition, it has been demonstrated that 2-AG reduced cell viability in a choriocarcinoma cell line and showed antiproliferative effects (24). In this study, we aimed to determine the function of DAGL enzymes in bioactive lipid metabolism in the human term placenta. Furthermore, we generated a profile of catalytically active serine hydrolases in placental tissue by using activity-based protein profiling (ABPP). Lipidomics, chemical proteomics, and *ex vivo* placental perfusion were applied to comprehensively study *in vitro* and *ex vivo* the effect of acute enzyme inhibition on placental lipid homeostasis.

## 2 Materials and methods

### 2.1 Experimental model and subject characteristics

Study was performed in accordance with the protocols approved by the ethical committee of the Medical University of Graz (Vote no: 29-319 ex 16/17 and 24-529 ex 11/12). All subjects gave written informed consent. Placentas from caesarean section and vaginal delivery were used within 20 min after delivery. Important subject characteristics of this study cohort are depicted in **Table 1**. In order to collect tissue samples, the placenta was divided into quadrants and a cross sectioned piece of 7-10 mm diameter was scissored from each quadrant. Tissue samples were either snap frozen in liquid nitrogen and stored at −80°C for protein isolation, or formalin fixed and embedded into paraffin for immunohistochemistry.

**Table 1:** Subject characteristics. Term placentas were collected from either caesarean section (CS) or vaginal deliveries (VD).

Term placentas, n=31		
Mode of birth (%)	CS	61.3
	VD	38.7
Gestational age	weeks ± days	39 ± 9
Placental weight [g]		616 (± 93.4)
Fetal sex (n)	Male	17
	Female	14
Fetal [g]/[cm]	weight	3312.4 (± 348.6)
	length	50.6 (± 2.1)
Maternal [kg/m^2^]	pre-pregnancy BMI	21.5 (± 2.4)
	BMI at delivery	26.8 (± 3)

Subjects with a pre-pregnancy recorded disease and/or BMI >26 kg/m2 were excluded. Values are depicted as mean (± SD); n represents number of placentas used in this study.

### 2.2 Quantitative real-time PCR

Frozen 20-30 mg placental tissue pieces were homogenized in 700μl Qiazol lysis reagent (Qiagen, Cat# 217004) for 20 seconds 6500 revolutions per minute, by MagnaLyser (Roche, Basel, Switzerland) followed by 1 minute on ice and repeated 3 times. Next, total RNA content from cells and tissue lysates were isolated using the RNeasy^®^Mini Kit (Qiagen, Cat# 217004). Reverse transcription was performed using 1μg of RNA and LUNA Script RT SuperMix Kit (New England Biolabs, Cat#E3010L). For RT-qPCR analysis LUNA Universal qPCR Master Mix (New England Biolabs, Cat#M3003E) and BioRad CFX384 Touch Syllabus were used. QuantiTect Primer Assays were used for gene amplification. For 18S reference gene amplification custom DNA oligos were designed (F-(5 ‘-3 ‘) CTACCACATCCAAGGAAGCA/R-(5 ‘-3 ‘) TTTTTCGTCACTACCTCCCCG). The expression of target genes DAGLα (GeneGlobe ID - QT00038164) and DAGLβ (GeneGlobe ID - QT00074319) was normalized to reference genes 18S, RPL30 (GeneGlobe ID - QT00056651) and HPRT1 (GeneGlobe ID - QT00059066). Target and reference gene ΔCT values are corrected for respective primer efficacy.

### 2.3 DAGLα/β *in situ* hybridization

To detect and discriminate DAGLα and DAGLβ mRNA on cellular level, RNAscope^®^ 2.5 HD Reagent Kit-RED assay (Advanced Cell Diagnostics, Cat#PN 322350) was used according to the manufacturer’s protocol. In short, 5 µm thick formaldehyde-fixed paraffin-embedded sections were de-paraffinized and pre-treated under standard pre-treatment conditions with hydrogen peroxide, target retrieval reagents and protease solution. The sections were covered with probe solution and incubated for 2 hours at 40°C using the HybEZ Hybridization System (Advanced Cell Diagnostics, Cat#PN 321710/321720). The sections were treated with AMP 1 to 6 according to the manufacturer´s manual, using the HybEZ Hybridization System. The multi-step hybridization process included hybridization to alkaline phosphatase-labeled probes and resulted in the detection of signal using Fast Red as a substrate. To combine ISH with immunohistochemistry (IHC), after performing ISH IHC was started from the blocking step as described below.

### 2.4 Immunohistochemistry

Placental tissue sections were blocked with 4% BSA and 10% secondary antibody host serum in PBS/0.3% Triton X100 and incubated overnight with primary antibody solutions. Primary antibodies for cytokeratin 7 (1:500, Abgent, Cat#AJ1229a), CD163 (1:200, Thermo Fisher Scientific, Cat#MA1-82342), and Von Willebrand Factor (1:500, Dako, Cat#A0082) were used. To detect Cytokeratin 7 (CK7) and Von Willebrand Factor (VWF) goat anti-rabbit Alexa Fluor 647 secondary antibody was used (1:500, Cell Signaling Technology, Cat#4414) and displayed in white. CD163 primary antibody incubation was followed by goat anti- mouse Alexa Fluor 488 secondary antibody application (1:500, Invitrogen, Cat#A32723) and displayed in green. Sections were counterstained with DAPI, sealed with a coverslip using VECTASHIELD^®^ Antifade Mounting Medium with DAPI (Vector Laboratories, Cat# H-1200-10) and stored at 4°C until imaged. Representative images were captured on Nikon A1 confocal microscope (original magnification ×40) and prepared using FIJI software v.1.51h.

### 2.5 Microscopy and signal quantification

For quantitative determination and localization analysis, ten z-stacks of each section were acquired using a Nikon A1 confocal with a ×40 objective at a step size of 0.5 µm. An automated image analysis was created with the software package FIJI v.1.51h. The analysis entailed a basic pre-processing, generating a maximum intensity projection and mean filter smoothing, followed by application of an algorithm-based threshold. Feature detection of channels containing CK7 or ISH information was achieved employing RenyiEntropy (25), IsoData for the CD163 channel (26) and Otsu for the VWF containing channel (27). Generated regions of interest (ROI) of the ISH were separated by watershed and counted the ROIs of the remaining channels were used to determine the cell type specific ISH localization due to overlap.

### 2.6 Gel-based activity-based protein profiling

Gel-based ABPP experiments were performed as previously described (28). Frozen tissues were thawed on ice and homogenized in cold lysis buffer (20 mM HEPES pH 7.2, 250 mM sucrose, 1 mM MgCl2, 2.5 U/mL benzonase). After incubation on ice for 15 min, tissue debris was pelleted by centrifugation (2500 × g, 3 min, 4°C) and supernatant was transferred to a clean tube. Subsequently the supernatant was centrifuged at 30.000 × g (90-120 min, 4°C) to pellet the membrane-associated fraction and separate it from the soluble proteome. After removal of the soluble supernatant, the membrane pellet was washed with cold HEPES buffer (20 mM, pH 7.2) followed by resuspension in cold HEPES buffer by pipetting. Concentration of membrane-associated and soluble proteome was quantified (Bradford; BioRad Technologies, CA, USA) and adjusted to desired concentration (2 mg/mL) in HEPES buffer (20 mM, pH 7.2). For direct labeling, proteomes were sequentially treated with one-step activity-based probes DH379 (30 min, 1 µM, Cy3, RT) and FP-Bodipy (15 min, 500 nM, Cy2, RT) or MB064 (30 min, 250 nM, Cy3, RT) alone in a 15 µL total reaction volume. For competitive ABPP experiments, this step was preceded by incubation with DH376 *in vitro* or *ex vivo* and LEI-105 *in vitro* at indicated concentrations. The reactions were quenched by the addition of 5 µL 4x Laemmli-buffer (BioRad Technologies, CA, USA). After separation by SDS-PAGE (10% acrylamide) at 180V for 75 min, samples were visualized by in-gel fluorescence scanning (Cy2 532/28, Cy3 605/50, Cy5 700/50 filter settings) using a flatbed fluorescent scanner ChemiDoc™ MP Imaging System (Bio-Rad, Hercules, CA, USA). Coomassie staining was used to control the protein loading. Gel fluorescence is shown in greyscale, and optical density of the signals was determined using ImageLab 6.1 Biorad.

### 2.7 DAGLβ activity assay

Membrane fractions of placental tissues were prepared as described above (see Gel-based activity-based protein profiling (ABPP)). Membrane lysates were diluted to 10 ng/µL in assay buffer (50 mM HEPES pH 7.5, 0.0025% Triton X-100). Fluorescent measurements were carried out at RT in a black flat bottom 96-well plate (Thermo Fisher Scientific, MA, USA) in the presence of 0.5 µM EnzChek™ lipase substrate (Thermo Fisher Scientific, Cat#E33955) in 100 μL final volume using a Clariostar plate reader (BMG Labtech, Germany) and excitation/emission wavelengths of 477/525. For competitive experiments placental membrane lysates were pre-incubated with DH376 (100 nM) and LEI-105 (1 µM) for 30 min at RT, respectively. DMSO served as vehicle control and denatured samples (1% SDS, 5 min, 100°C) served as background controls. Background substrate hydrolysis was deducted from each measurement. Each data point is the mean of three technical replicates of n=3 placentas, for concentration testing and n=4 placentas for competitive experiments. The slope t=10 min to t=60 min was used as the enzymatic rate (RFU/min). Enzyme kinetics were plotted as curves in Graph Pad Prism 9 Software (GraphPad Software Inc., CA, USA).

### 2.8 Chemical proteomics with label-free quantification

Placental tissues were homogenized and prepared as described in Gel-based activity-based protein profiling (ABPP) section. The chemical proteomics workflow is based on previously published protocol (29) and conducted with minor modifications. In short, cytosolic and membrane fractions of placental tissue lysates (250 µg protein, 1 mg/mL, n=5) were incubated with serine hydrolase probe cocktail (10 µM MB108, 10 µM FP-Biotin, 30 min, 37 ˚C, 300 rpm). A pool of denatured vehicle control samples (1% SDS, 5 min, 100°C) was taken along as a negative control. Following steps were preformed according to protocol, including precipitation, alkylation, avidin enrichment, on-bead digestion, and sample preparation. Dried and desalted peptide samples were stored at -20°C until LC-MS analysis. Prior to measurement, samples were reconstituted in 50 µL 97:3:0.1 solution (H2O, ACN, FA) containing 10 fmol/µL yeast enolase digest (Waters, cat# 186002325) and transferred to LC-MS vials. Additionally, a quality control sample was prepared to prevent overloading the nanoLC system and the automatic gain control (AGC) of the QExactive HF mass spectrometer. LC-MS data was analyzed by MaxQuant software 2.0 applying match between runs. For further analysis, the following cut-offs were used: unique peptides ≥ 2, identified peptides ≥ 2, ratio positive over negative control ≥ 2. Additionally, targets were filtered against a putative probe-target list including human metabolic serine hydrolases.

### 2.9 *Ex vivo* placental perfusion

Placental perfusion setup is based on the setup published by Schneider et al. (30) and adapted as published by Hirschmugl et al. (31). In short, within 30 min after delivery of the placenta a single placental cotyledon chorionic-artery and vein pair was cannulated and flushed with perfusion buffer, containing DMEM (DMEM, phenol red free, Gibco by Life Technologies, ThermoFisher Scientific, MA, USA) mixed (3:1) with Earl's buffer (6.8 g/L NaCl, 0.4 g/L KCl, 0.14 g/L NaH2PO4, 0.2 g/L MgSO4•7H2O, 0.2 g/L CaCl2, 2.2 g/L NaHCO3, pH 7.4, all Merck, Darmstadt, Germany), amoxicillin (250 mg/L, Sigma-Aldrich, Steinheim, Germany), glucose (2 g/L Merck, Darmstadt, Germany), and essential fatty acid free bovine serum albumin (BSA) (35 g/L, Sigma-Aldrich, Seinheim, Germany). The cannulated cotyledon and surrounding tissue were placed in the pre-warmed perfusion chamber and the fetal circulation was connected to a magnetic pump (Codan, Salzburg, Austria) with a constant fetal artery inflow of 3 mL/min. The perfusion buffer is constantly fumigated by a gas exchange device (LivingSystems, St. Albans, VT, US) operated with 95% N2 and 5% CO2 on the fetal site during the experiment. A micro catheter pressure sensor (Millar, US) inserted into the fetal arterial cannula recorded the backflow pressure which should not exceed an average of 65 mbar. The impermeability of the perfused cotyledon was monitored within the first 30 min and each cotyledon displayed at least 95% fetal flow recovery. The maternal circulation was established by inserting three rounded needles into the intervillous space of the cotyledon with a flow rate of 9 mL/min. During the experiment, the perfusion buffer was gassed with 5% CO_2_, 20% O_2_ and 75% N_2_ through the gas exchanging device. DH376 (1 µM) was added to the fetal and maternal perfusion buffer reservoirs and the system changed to closed circuit in all inhibitor experiments. During the experiment maternal and fetal perfusates were collected every 30 min *via* a sampling port and oxygen (pO_2_), carbon dioxide (pCO_2_), pH, lactate production, and glucose consumption measurements were applied by a blood gas analyzer (Radiometer, Copenhagen, Denmark). The data sets obtained by the blood gas analyzer, magnetic pumps, and pressure sensor were registered and recorded *via* LabVIEWbased recording software (Beko engineering, Graz, Austria). After 4h of closed perfusion time, samples of both circuits were collected, centrifuged, and stored at −80°C. The perfused placental tissue was processed in cold PBS and snap frozen in liquid nitrogen until further analysis.

### 2.10 Lipid analysis by LC-MS

Placenta (pl, ~10 mg powdered), and perfusate (pf, 140 µl) samples were extracted according to Matyash et al. (32). In brief, samples were homogenized using two beads (stainless steel, 6 mm) on a Mixer Mill (Retsch, Haan, GER; 2x10sec, frequency 30/s) in 700 µl methyl-tert-butyl ether (MTBE)/methanol (3/1, v/v) containing 500 pmol butylated hydroxytoluene, 1% acetic acid, and internal standards (IS; pl: 20 pmol 15:0/15:0/15:0 triacylglycerol, 13 pmol rac-17:0/17:0 diacylglycerol, rac-17:0 monoacylglycerol, 50 pmol 17:0/17:0 phosphatidylcholine, Larodan, Solna, Sweden; 133 pmol 17:0/17:0 phosphatidylethanolamine, 30 pmol 17:0/17:0 phosphatidylserine, 8 pmol 17:1 lyso-phosphatidylcholine, 30 pmol 17:1 lyso-phosphatidylethanolamine, Avanti Polar Lipids, Alabaster, AL, USA; cb and pf: 2 nmol 17:0 FA, 800 pmol C21:0 FA, Sigma-Aldrich, St. Louis, MO, USA). Total lipid extraction was performed under constant shaking for 30 min at RT. After addition of 140 µl dH_2_O (pl) and further incubation for 30 min at RT, samples were centrifuged at 1,000 x g for 15 min. 500 µl of the upper, organic phase were collected and dried under a stream of nitrogen. Lipids were resolved in 500 µl MTBE/methanol (3/1, v/v). Pl extracts were diluted 1:4 in 2-propanol/methanol/dH_2_O (SolA; 7/2.5/1, v/v/v) for LC-MS analysis. To determine fatty acid levels, 200 µl (pf) were derivatized according to Bollinger et al. (33) using the AMP+ MS Kit (Cayman Chemical, Michigan, USA) and resolved in 500 µl SolA for LC-MS analysis. Protein precipitates of the extractions were dried, solubilized in NaOH (0.3 N) at 65°C for 4 h and the protein content was determined using Pierce™ BCA reagent (Thermo Fisher Scientific, MA, USA) and BSA as standard. Chromatographic separation was performed on a 1290 Infinity II LC system (Agilent, CA, USA) equipped with Zorbax RRHD Extend-C18 column (2.1x50 mm, 1.8 µm; Agilent, CA, USA) running a 10 min linear gradient from 60% solvent A (H2O; 10 mM ammonium acetate, 0.1% formic acid, 8 µM phosphoric acid) to 100% solvent B (2-propanol; 10 mM ammonium acetate, 0.1% formic acid, 8 µM phosphoric acid). The column compartment was kept on 50°C. A 6470 Triple Quadrupole mass spectrometer (Agilent, CA, USA) equipped with an ESI source was used for detection of lipids in positive mode. Data acquisition was done by MassHunter Data Acquisition software (B.10, Agilent, CA, USA) either in MRM (glycerol- and glycerophospholipids) or SIM (fatty acid derivatives) mode. Lipidomic data were processed using MassHunter Workstation Quantitative Analysis for QQQ (V.9, Agilent, CA, USA), normalized for recovery, extraction-, and ionization efficacy by calculating analyte/IS ratios (AU) and expressed as AU/µg protein.

### 2.11 Statistical analysis

Graph Pad Prism 9.02 Software (GraphPad Software Inc., CA, USA) was used for statistical analysis and graph plotting. Data are presented as mean ± SEM. All obtained datasets were tested for normal distribution with the Shapiro-Wilk and Kolmogorov-Smirnov test. Depending on the distribution of datasets parametric or non- parametric statistical tests were applied. If two or more normal distributed groups were compared student`s t-test or one-way ANOVA, including Benjamini- Hochberg *post-hoc* was performed. If the dataset did not show a normal distribution Mann-Whitney U test or Kruskal-Wallis test followed by Benjamini- Hochberg *post hoc* was applied. Two-way ANOVA was applied comparing two or more groups including different variables using Benjamini- Hochberg *post hoc* for multiple comparison correction. All herein presented p-values correspond to a FDR of 1% for multiple testing and p-values below 0.05 were considered statistically significant.

## 3 Results

### 3.1 Detection of DAGL mRNA and activity in placental tissue lysates

**Figure 1:**
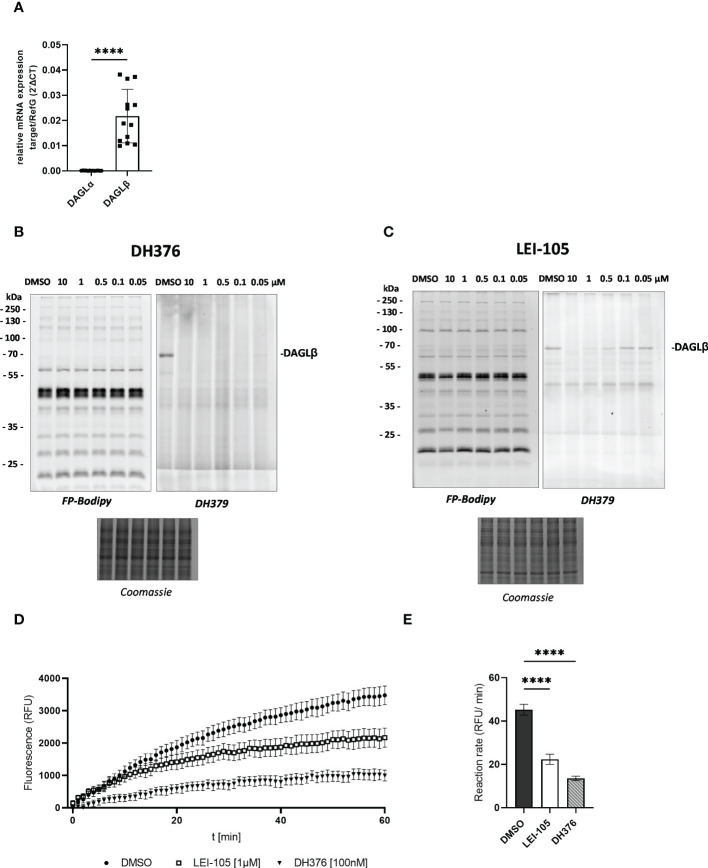
Detection of DAGL mRNA and activity in placental tissue lysates. **(A)** Relative DAGLα/β target gene mRNA levels were detected by RT-qPCR. Results were normalized to reference genes (RefG) 18S, RLP30 and HRPT1 detected in each sample and calculated as ΔCT. For statistics student`s t-test was applied and ΔCT values are depicted as 2 -ΔCT (n=13). **(B)**, **(C)** Visualization of DAGLβ activity and selectivity profile of DH376 and LEI-105 using in-gel ABPP. Placental membrane proteomes were profiled by competitive ABPP using a probe cocktail of FP-Bodipy [500 nM] (Cy2, green) and DH379 [1 µM] (Cy3, red). Samples were incubated with indicated inhibitor concentrations or DMSO as a vehicle control. Concentration-dependent inhibition of DAGLβ by DH376 **(B)** and LEI-105 **(C)**. Coomassie staining served as a protein loading control. **(D)** Hydrolase activity was determined using EnzChek™ lipase substrate [0.5µM] and displayed as relative fluorescence units (RFU) per time. DAGLβ activity determined by applying LEI-105 [1 µM] and DH376 [100 nM]. **(E)** The slope of the linear interval t=10 to t=60 min was used to calculate the enzymatic rate (RFU/min). One-way ANOVA for multiple comparisons followed by Benjamini- Hochberg *post hoc* was applied to quantify the differences of enzymatic activities (n=4). Data are depicted in mean ± SEM; ****p ≤ 0.0001.

To elaborate DAGL expression in placental tissues, we first determined DAGLα/β mRNA levels by RT-qPCR. In comparison to DAGLα substantially higher DAGLβ expression was observed (**Figure 1A**, p<0.0001). Serine hydrolase activities in the placenta were examined by gel-based ABPP. To obtain a broad view of placental serine hydrolase activities and DAGL-specific signals, we used a probe cocktail of non- selective FP-Bodipy (34) and the DAGLα/β directed fluorescent probe DH379 (28). Using FP-Bodipy, we detected a broad spectrum of active enzymes in placental lysates. Application of DH379 visualized DAGLβ at the expected molecular weight of ~70 kDa (**Figures 1B, C**). To confirm the presence of active DAGLβ, competitive ABPP was applied using the DAGL inhibitors DH376 (IC_50_ 3–8 nM) (28) and LEI-105 (IC_50_ ~32 nM) (35). Application of DH376 and LEI-105 reduced DAGLβ activity in a dose-dependent manner. DH376 led to full inhibition at the lowest concentration of 0.1 µM (**Figure 1B**), while LEI-105 treatment led to a substantial reduction of DAGLβ signals at a concentration of 0.5 µM (**Figure 1C**). Notably, we were not able to detect DAGLα activity at the expected molecular weight of ~120 kDa, using the DAGLα tailored probe MB064 (36), likely due to low expression levels compared to DAGLβ (**Supplementary Figure 1**). DAGLβ activity in placenta tissue lysates was also investigated using the commercially available lipase substrate EnzChek™ (**Figure 1D**). We applied 100 nM of DH376, which represented the lowest inhibitor concentration leading to potent enzyme inhibition of DAGLβ in-gel (**Figure 1B**). In comparison to DH376, LEI-105 exhibits lower activity against DAG-lipases and acts as a reversible enzyme inhibitor. Although a substantial reduction in DAGLβ enzyme activity was observed by application of 0.5 µM (**Figure 1C**), we applied 1 µM LEI-105 to ensure complete enzyme inhibition over time. The application of LEI-105 and DH376 reduced hydrolase activity by 51% and 70%, respectively (**Figure 1E**). The differences in inhibitor efficacy can be explained by previous observations showing that LEI-105 exhibits higher selectivity for DAGLβ than DH376. At the used inhibitor concentration, DH376 is expected to inhibit α/β hydrolase domain-containing protein 6 (ABHD6), carboxylesterase 1 and 2 (CES1/2), and hormone- sensitive lipase (HSL) (28), which can contribute to the hydrolysis of the EnzChek™ substrate. Gel-based ABPP experiments enabled us to detect specific DAGLβ activity in placental tissue and we could further decipher DAGL-dependent substrate hydrolysis by administration of DH376 and LEI-105. Overall, our observations indicate that the placenta predominantly expresses DAGLβ, which consequently can affect DAG, MAG, and fatty acid (FA) metabolism.

### 3.2 Activity profiling of placental metabolic serine hydrolases

**Figure 2:**
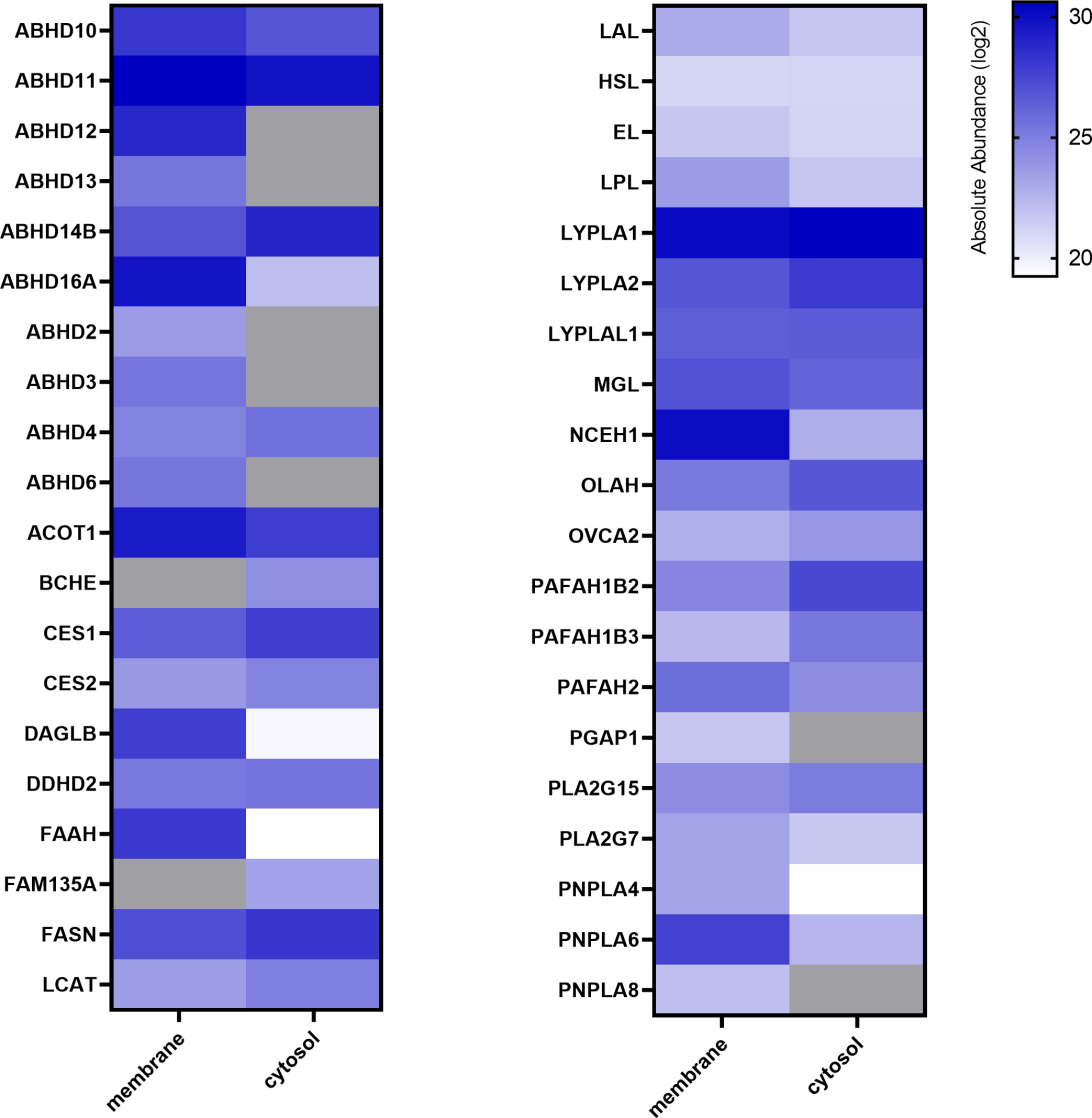
Activity profiling of placental metabolic serine hydrolases. Membrane and cytosolic tissue protein fractions were labeled with hydrolase probe cocktail (MB108, FP-Biotin [10 µM]) and analyzed by chemical proteomics. Absolute abundance refers to the mean of LFQ intensities of vehicle perfused placentas and is depicted in alphabetical order as heat map (blue scale, log2), not detected proteins are depicted in grey (n=5).

ABPP using FP-Bodipy already suggested that the placenta expresses a broad spectrum of serine hydrolases (**Figures 1B, C**). To get a profile of these enzymes, we performed mass spectrometry-based chemical proteomics utilizing the biotinylated non- selective probes MB108 and FP-Biotin for target identification. While gel-based ABPP experiments strongly rely on specific inhibitors for target identification, MS- based ABPP enables target enrichment and provides high sensitivity. To increase the resolution of proteins, tissue lysates were separated into membrane and cytosolic fraction. This approach resulted in the identification of 38 and 33 different serine hydrolases in membrane and cytosolic fractions, respectively (**Figure 2**). Activities of several α/β hydrolase domain-containing protein family members (ABHD) and phospholipases such as DDHD2, patatin-like phospholipase domain-containing proteins (PNPLA) and members of the phospholipase A2 family (PLA2) were identified. Furthermore, lipases involved in DAG, MAG and FA metabolism, including HSL, CES1/2 and acyl-coenzyme A thioesterase (ACOT1) were detected. Within the 2-AG biosynthetic active enzymes, we again exclusively detected DAGLβ activity. Taken together, performing chemical proteomics allowed us to generate an overview of the lipolytic proteome of human placental tissue and demonstrated a broad spectrum of metabolic hydrolase activities.

### 3.3 DAGLβ expression is mainly confined to trophoblasts

**Figure 3:**
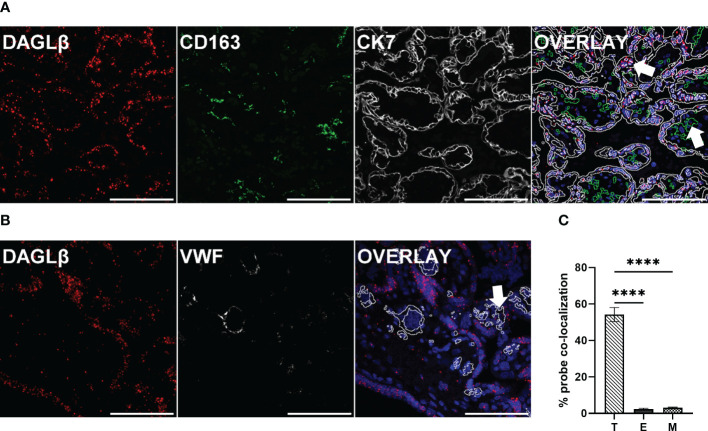
DAGLβ expression is mainly confined to trophoblasts. DAGLβ transcripts were detected in placental tissues using RNAscope^®^ 2.5 HD RED assay. **(A)** DAGLβ transcripts detected in CK7 positive trophoblasts and CD163 positive placental macrophages. **(B)** DAGLβ mRNA was localized to VWF stained endothelial cells. Nuclei were counterstained with DAPI (blue). Arrowheads in merged micrographs indicate probe co-localization to different cell types. Magnification x40, Scale bar 100 µm. For quantitative determinations, ten images of four individual placentas were captured on Nikon A1 confocal microscope. Probe co-localization was quantified by Fiji software. **(C)** Relative distribution of DAGLβ transcripts to trophoblasts (T), endothelial cells **(E)** and placental macrophages (M) based on ISH signals (n=4). Statistical analysis was performed using one- way ANOVA, followed by Benjamini- Hochberg *post hoc* test (n=4). Representative stainings are shown in **(A)** and **(B)**. Data are depicted in mean ± SEM; ****p < 0.0001.

As the human placenta is composed of different highly specialized cell types, we aimed to determine the localization of DAGLβ *in situ*, by using specific RNA probes. To localize the transcripts to distinct cell types of the placenta, immunofluorescence (IF) was applied. The visualization of DAGLβ transcripts was combined with cytokeratin 7 (CK7) staining, representing trophoblasts, which are building up the first structural barrier between maternal and fetal compartment and CD163 as a pan macrophage marker (**Figure 3A**). To identify feto-placental endothelial cells, which are lining placental vessels and are in direct contact with fetal blood, Von- Willebrand Factor (VWF) was applied (**Figure 3B**). We localized DAGLβ transcripts mainly to CK7 positive trophoblasts (**Figure 3A**). Quantitative analysis of the signals revealed that 54% of DAGLβ mRNA was localized to trophoblasts (T), while negligible signals of 2% and 3% were detected in endothelial cells (E) and macrophages (M), respectively (**Figure 3C**). In contrast to DAGLβ, we could not detect clear signals for DAGLα, confirming our observation that this enzyme is poorly expressed in the human placenta (**Supplementary Figure 2**).

### 3.4 Inhibition of DAGLβ activity leads to reduced DAG, MAG and FA levels in perfused placental tissue

**Figure 4:**
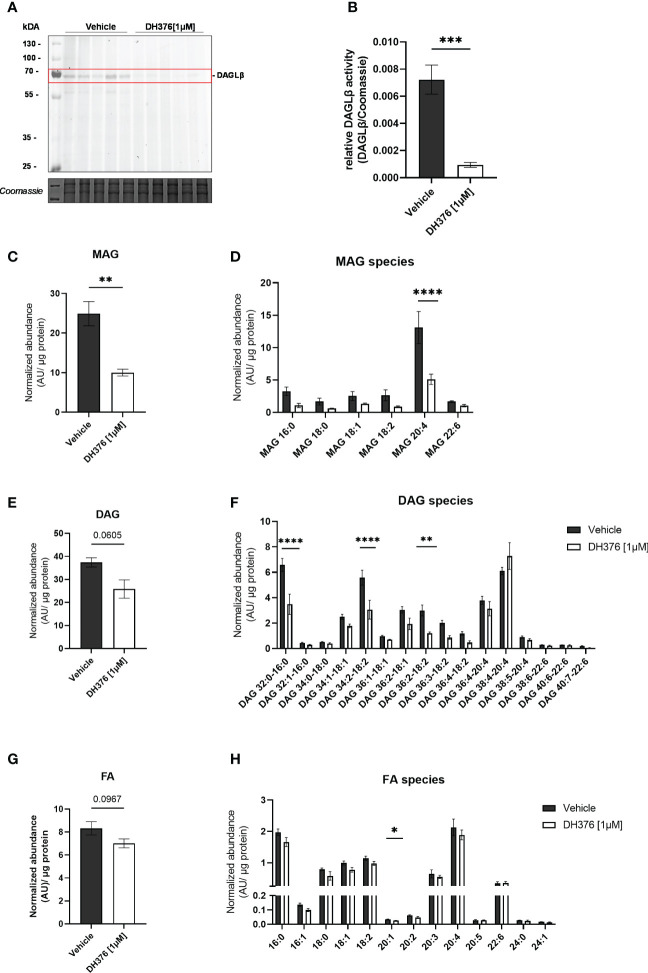
Inhibition of DAGLβ activity leads to reduced DAG, MAG and FA levels in perfused placental tissue. **(A)** *In vitro* labeling of enzymes in DH376 [1 µM] and vehicle perfused placental membrane proteomes by direct ABPP using DH379. **(B)** Densiometric quantification of in gel ABPP demonstrated significantly decreased DAGLβ activity in DH376 perfused placentas compared to vehicle controls. Student´s t-test was performed for statistical testing (n=5). **(C)** Total monoacylglycerol (MAG) tissue levels in DH376 [1 µM] and vehicle perfused tissues. **(D)** Depiction of all measured MAG species by LC-MS. **(E)** LC-MS analysis of total diacylglycerol tissue levels (DAG) and diacylglycerol species in vehicle and DH376 perfused placental tissues **(F)**. **(G)** Total tissue FA levels and corresponding FA species **(H)**. Lipid levels are expressed as arbitrary units (AU) and were normalized to total tissue protein (µg). Student´s t-test and multiple t-test followed by Benjamini- Hochberg *post hoc* was performed, respectively (n=3 lipid levels, n=5 FA levels). Data are depicted as mean ± SEM; *p ≤ 0.05, **p ≤ 0.01, ***p ≤ 0.001, ****p< 0.0001.

To better understand the specific function of DAGLβ in the intact organ, the lipid profile was examined after tissue perfusion with/out inhibitor. First, we examined the extent of DAGLβ inhibition obtained after perfusion by in-gel ABPP. Using DH379, we again identified the DAGLβ band (~70 kDa) in the vehicle perfused proteome (**Figure 4A**; **Supplementary Figure 3**). By co-application of DH376 [1µM], signal intensity was strongly reduced by 87%, confirming target engagement (**Figure 4B**). Thus, our results suggest that the application of DH376 at a concentration of 1 µM in an *ex vivo* setting leads to substantial inhibition of DAGL, which may also be accompanied by changes in the lipid profile. To examine the consequences of DAGLβ inhibition under the applied conditions, we analyzed changes in lipid species in perfused placental tissue. In accordance with the proposed cellular function of DAGL, total MAG levels were decreased by 60% (**Figure 4C**), resembled by a reduction of all detected MAG species (**Figure 4D**). Notably, the obtained data further demonstrated significant decreased 2-AG levels (MAG 20:4) upon inhibition of DAGLβ (**Figure 4D**). Interestingly, decreased MAG levels were not accompanied by augmented DAG concentrations, but a trend to decreased levels could be detected (**Figure 4E**). In fact, we observed significant reductions in specific DAG species, including DAG 32:0-16:0, DAG 34:2-18:2 and DAG 36:2-18:2 (**Figure 4F**). Furthermore, FA concentrations showed a downward trend in inhibitor perfused tissues (**Figure 4G**), of which eicosenoic acid (FA 20:1) was significantly reduced, indicating an effect on the hydrolysis of *sn-1* FA of DAGs (**Figure 4H**). In contrast, FA levels in the maternal and fetal circuit remained unchanged (**Supplementary Figures 4A, B**).

## 4 Discussion

DAG lipases occupy a central role in multiple lipid signaling pathways by regulating DAG (37), endocannabinoid levels and downstream inflammatory mediators (4, 28). Studies in mice demonstrated that acute blockade of DAGL by DH376 *in vivo* led to significant reductions in endocannabinoid-, AA-, and prostaglandin levels in the central nervous system (28). Moreover, it has been shown that pharmacological inhibition and genetic disruption of DAGLα/β suppress induction of 2-AG and prostaglandin levels upon lipopolysaccharide treatment, which was accompanied by decreased pro-inflammatory cytokine secretion (4, 5, 28). Recently, Shin et al. identified DAGLβ as polyunsaturated fatty acid- specific triacylglycerol lipase by demonstrating robust hydrolysis activity for triarachidonin (C20:4 FA) or tridocosahexaenoin (C22:6 FA) *in vitro* (6). Hence, understanding the role of these versatile enzymes in human physiology and disease gained considerable interest. Here, we aimed to study the role of DAGL in placental lipid homeostasis.

ABPP assays were used to screen for serine hydrolase activities in the human placenta and particularly to examine the functional state of DAGL enzymes. The use of a fluorescent DAGL-tailored probe enabled us to detect DAGLβ activity at the corresponding molecular weight of ~70 kDa. Competitive ABPP with selective inhibitors confirmed that the signal was DAGLβ. DH376 has been described as potent, central active and covalent DAGL inhibitor (IC_50_ of 3-8 nM) (28). Since it has been reported that this compound cross-reacts with several other lipases such as CES1/2, HSL and ABHD6, we decided to include LEI-105 to verify our findings. LEI-105 is described as highly selective, but reversible DAGL inhibitor (IC_50_ ~32 nM) (35). Importantly, it has been shown that this compound did not affect the activity of other endocannabinoid-related hydrolases such as ABHD6 (35). Complete enzyme inhibition of placental DAGLβ could be achieved by applying relatively high inhibitor concentrations, as LEI-105 represents a non-covalent inhibitor and shows ~4-fold lower activity against DAGL enzymes compared to DH376. In conclusion, administration of LEI-105 validated our observations in-gel and contributed to the determination of DAGL- dependent substrate hydrolysis. Conversely, DAGLα activity was not detectable by ABPP, suggesting that DAGLβ is the principal active DAG-lipase in the human term placenta. The predominance of DAGLβ over α in placental tissue was further corroborated on transcriptional level *in vitro* as well as *in situ*. These findings are in accordance with previous studies showing that DAGLβ is mainly found in peripheral metabolically active tissues such as the liver, where DAGLβ-/- mice showed 90% reductions in 2-AG levels (2). As placental tissue is composed of different cell types, we specifically looked at the spatial expression of DAGLβ. DAGLβ transcripts were mainly located to CK7-postive trophoblasts, lining the first cellular barrier between the maternal and fetal circulation. Co-localization of DAGLβ to trophoblasts, which reflect the main site of action upon maternally derived signals, is in concordance with the expression sites of other lipid related enzymes in this organ (38).

In order to assess the importance of DAGL in the spectrum of the lipolytic enzymes in placental tissue we further generated an activity- based profile of serine hydrolases. The chemoproteomic analysis revealed a broad spectrum of hydrolases, which determine lipid metabolism and signaling. Within the 2-AG biosynthetic enzymes we again exclusively detected DAGLβ. Further, specific activity of enzymes involved in degradation of 2-AG, such as MGL, fatty acid amide hydrolase (FAAH), ABHD6 and ABHD12 was detected. It has been shown that beside MGL, which is the main enzyme for 2-AG hydrolysis, ABHD6/12 and FAAH also possess 2-AG hydrolytic activity (39, 40). Blankman et al. suggested that the simultaneous occurrence of different 2-AG hydrolytic enzymes could be explained by the regulation of distinct subcellular 2-AG pools. In accordance with previous observations, ABHD6/12 were exclusively identified in placental membrane preparations, whereas MGL activity was found in the cytosolic and membrane fraction (40). Interestingly, ABHD12 showed the highest activity in our dataset, compared to other 2-AG metabolizing enzymes. The major function of ABHD12 is the hydrolysis of lysophosphatidylserine as shown by genetic depletion in mice (41). The specific role of this enzyme in the placenta remains to be investigated, since only descriptive data is available yet (22). Besides 2-AG, anandamide (AEA) was one of the first discovered endocannabinoids and FAAH is the main catabolic enzyme in this pathway (42). Moreover, ABHD4 activity was detected, which contributes to AEA biosynthesis. In addition, several hydrolases determining lipid and FA metabolism, including (lyso)phospholipases, HSL and CES1/2 were identified.

This study set out with the aim of assessing the importance of DAGLβ activity in the lipid homeostasis of the human term placenta. Therefore, we looked at the functional consequences of pharmacological enzyme inhibition *ex vivo*, by applying DH376 as an inhibitor targeting DAGL activity. Lipidomic analysis of perfused tissue samples showed that acute inhibition of DAGLβ led to significantly reduced total MAG tissue levels, confirming the well-described role of DAGL in DAG catabolism. In fact, we could observe a significant decrease in 2-AG levels and a trend towards reduced saturated as well as mono- and polyunsaturated MAG species. In contrast to previously published data, the decrease in MAG levels was not followed by an increase in respective DAGs, suggesting that DAGs are efficiently metabolized in the absence of DAGL. Interestingly, specific DAG species showed a significant reduction upon DAGLβ inhibition, indicating that the enzyme may possess hydrolase activity against TAGs. In this context, DAGLβ has been previously described as polyunsaturated- specific TAG lipase in mice using genetic and pharmacological approaches (6). The decline in eicosenoic acid (FA 20:1), in inhibitor perfused tissues, suggests that DAGLβ prefers 20:1 species at *sn-1* position of DAGs. Interestingly, we could not detect considerable differences of FA levels neither in the maternal nor in the fetal circulation. In contrast, *in vivo* pharmacological inhibition of DAGLα/β by DH376 led to significant reductions of AA levels in murine central nervous tissues (28). Unchanged AA levels could be explained by compensatory or bypass activities ensuring a constant supply of polyunsaturated fatty acids to the fetus. Furthermore, Hirschmugl et al. demonstrated that only a small proportion of free FA are directly transferred across the placenta and emphasized the tightly regulated release of FA out of metabolic pools of the placenta (31). It is also important to note that DAGLβ is predominantly expressed in trophoblasts and lipid extracts were obtained from whole tissue. Nonetheless, we observed a substantial decrease in MAGs indicating that DAGLβ activity strongly affects lipid homeostasis distinctively in this specific placental cell type.

This study describes for the first time that 2-AG is dramatically reduced after DAGL inactivation in the human placenta. Since 2-AG represents only one of the main endocannabinoids, this study is limited by the lack of information on the potential simultaneous regulation of AEA. Although, both endocannabinoids exhibit distinct synthesis, transport and degradation processes, one may speculate that alterations in lipid levels affect concomitant lipid signaling pathways, and compensatory or bypass mechanisms could be activated to restore lipid homeostasis. In particular, a potential mechanism for synaptic crosstalk and feed-back regulatory mechanisms between the two pathways has already been described in tissues of the central nervous system (43, 44). Furthermore, only one inhibitor concentration and experimental timepoint was used for *ex vivo* experiments. Since DAGLα exhibits a short half- life (< 4 h) and cumulative evidence supports the on-demand model of endocannabinoid biosynthesis (28, 45), it would be of great interest to study the consequences of enzyme blockage on the dynamic composition of placental lipids over time.

In summary, our study demonstrates that the application of small molecule inhibitors in perfusion experiments provides a very useful tool to investigate enzyme function as close as possible to the *in vivo* situation. In addition, ABPP is a powerful technique to visualize the active pool of enzymes and confirm target engagement in such *ex vivo* tissue experiments. The integration of these two approaches provided evidence that inhibition of DAGLβ affects tissue lipid homeostasis with no direct effect on the FA profile in the maternal or fetal compartment. We expect that further experiments by utilizing serine hydrolase inhibitors will strongly improve our understanding of the role of these enzymes in lipid signaling and metabolism at the maternal-fetal interface and reveal important insights related to placental function in normal and compromised pregnancies.

## Data availability statement

The mass spectrometry proteomics data have been deposited to the ProteomeXchange Consortium via the PRIDE (46) partner repository with the dataset identifier PXD039930.

## Ethics statement

The studies involving human participants were reviewed and approved by Ethical Committee of the Medical University of Graz, Graz, Austria. The patients/participants provided their written informed consent to participate in this study.

## Author contributions

Conceptualization, CW and NB. Methodology, NB, BH, TB, TW, TE and NF. Software, JG. Investigation, NB, TE and NF. Writing – Original Draft, NB, CW, BH and RZ. Funding Acquisition, CW. Resources, MS, RB-G and RZ. Supervision, CW, BH, TW, MS and RZ. All authors contributed to the article and approved the submitted version.
