# Supplementary material for: Inhibition of diacylglycerol lipase β modulates lipid and endocannabinoid levels in the ex vivo human placenta
Source: Front Endocrinol (Lausanne). 2023 Feb 14;14:1092024. doi: 10.3389/fendo.2023.1092024 (PMC9971001; doi:10.3389/fendo.2023.1092024)
Supplement: Supplementary file 1 [file DataSheet_1.pdf]

Supplementary Data

Supplementary Table 1. Identified metabolic serine hydrolases in placental tissue. Abbreviation and description are depicted as annotated on uniprot.org.

**Hydrolase**	**Description**
ABHD10	α/β-hydrolase domain (ABHD)-containing protein 10
ABHD11	α/β-hydrolase domain (ABHD)-containing protein 11
ABHD12	α/β-hydrolase domain (ABHD)-containing protein 12
ABHD13	α/β-hydrolase domain (ABHD)-containing protein 13
ABHD14B	α/β-hydrolase domain (ABHD)-containing protein 14B
ABHD16A	α/β-hydrolase domain (ABHD)-containing protein 16A
ABHD2	α/β-hydrolase domain (ABHD)-containing protein 2
ABHD3	α/β-hydrolase domain (ABHD)-containing protein 3
ABHD4	α/β-hydrolase domain (ABHD)-containing protein 4
ABHD6	α/β-hydrolase domain (ABHD)-containing protein 6
ACOT1	Acyl-coenzyme A thioesterase 1
BCHE	Cholinesterase
CES1	Carboxylesterase 1
CES2	Carboxylesterase 2
DAGLB	Diacylglycerol lipase beta
DDHD2	Phospholipase DDHD2
FAAH	Fatty acid amide hydrolase
FAM135A	Protein FAM135A
FASN	Fatty acid synthase
LCAT	Phosphatidylcholine-sterol acyltransferase
LAL	Lysosomal acid lipase/cholesteryl ester hydrolase
HSL	Hormone-sensitive lipase

EL	Endothelial lipase
LPL	Lipoprotein lipase
LYPLA1	Acyl-protein thioesterase 1
LYPLA2	Acyl-protein thioesterase 2
LYPLAL1	Lysophospholipase-like protein 1
MGL	Monoacylglycerollipase
NCEH1	Neutral cholesterol ester hydrolase 1
OLAH	S-acyl fatty acid synthase thioesterase
OVCA2	Esterase OVCA2
PAFAH1B2	Platelet-activating factor acetylhydrolase IB subunit alpha2
PAFAH1B3	Platelet-activating factor acetylhydrolase IB subunit alpha1
PAFAH2	Platelet-activating factor acetylhydrolase 2
PGAP1	GPI inositol-deacylase
PLA2G15	Phospholipase A2 group XV
PLA2G7	Platelet-activating factor acetylhydrolase
PNPLA4	Patatin-like phospholipase domain-containing protein 4
PNPLA6	Patatin-like phospholipase domain-containing protein 6
PNPLA8	Calcium-independent phospholipase A2-gamma

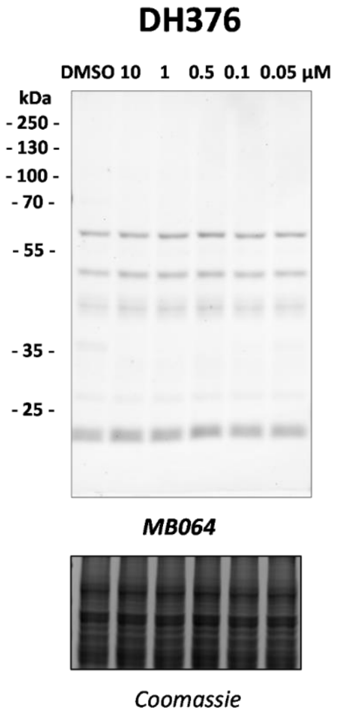

Supplementary Figure 1: Competitive ABPP using the DAGLα directed probe MB064. Placental membrane proteomes were profiled by competitive ABPP using MB064 [250 nM] and indicated inhibitor concentrations of DH376. Coomassie staining served as a protein loading control. Fluorescent gel images shown in grey scale.

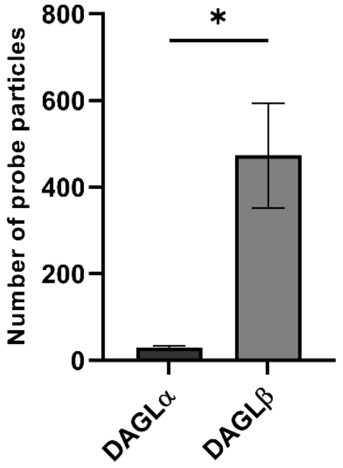

Supplementary Figure 2: DAGLβ expression predominates over DAGLα in the human placenta. DAGLα/β transcripts were detected in placental tissues using RNAscope® 2.5 HD RED assay. For quantitative determinations, ten images of four individual placentas were captured on Nikon A1 confocal microscope. Probe co-localization was quantified by Fiji software. Mann-Whitney U test was performed to quantify the number of DAGLα/β probe particles in placental tissue (n=4). Data are depicted in mean ± SEM; * $p \leq 0.05$

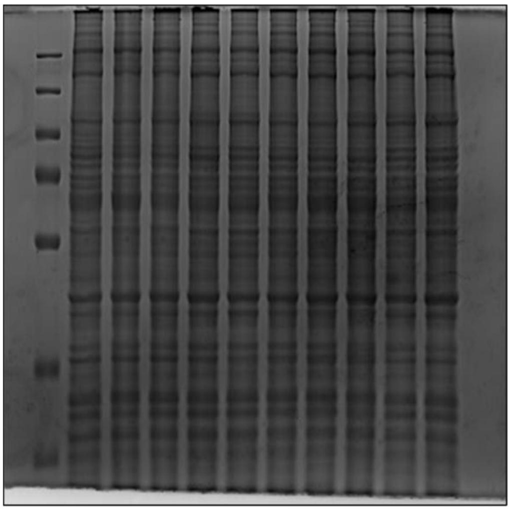

Supplementary Figure 3: Full size Coomassie image. Depiction of the full size image of the Coomassie section displayed in Figure 4A.

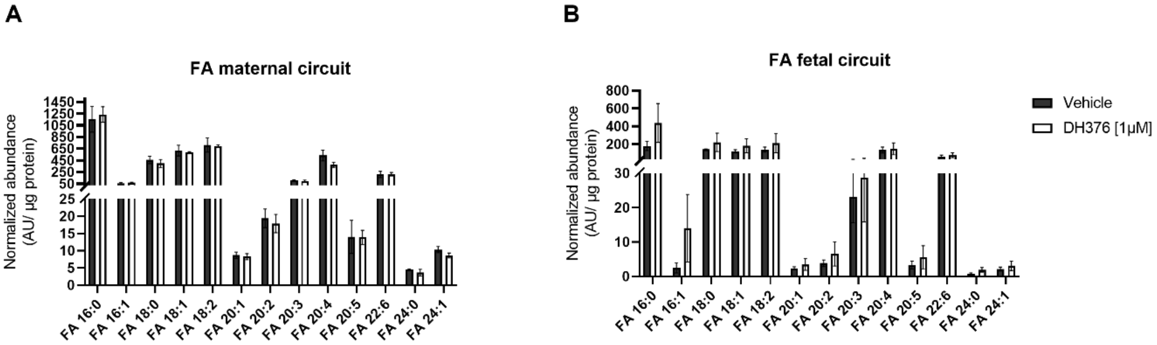

Supplementary Figure 4: LC-MS analysis of fatty acid (FA) levels were determined in maternal (A) and fetal circuits (B) of vehicle control and DH376 inhibitor perfusion experiments. Lipid levels are expressed as arbitrary units (AU) and were normalized to total tissue protein. For statistical testing multiple t-test followed by Benjamini- Hochberg post hoc was performed, respectively (n=3). Data are depicted in mean ± SEM
